# The Local Effects of Ovarian Diathermy in an Ovine Model of Polycystic Ovary Syndrome

**DOI:** 10.1371/journal.pone.0111280

**Published:** 2014-10-24

**Authors:** Fiona Connolly, Michael T. Rae, Mairead Butler, Alexander L. Klibanov, Vassilis Sboros, Alan S. McNeilly, W. Colin Duncan

**Affiliations:** 1 Medical Research Council Centre for Reproductive Health, University of Edinburgh, Edinburgh, United Kingdom; 2 School of Health, Life and Social Sciences, Edinburgh Napier University, Edinburgh, United Kingdom; 3 Institute of Biophysics, Biochemistry and Bio-Engineering, Heriot Watt University, Edinburgh, United Kingdom; 4 Department of Biomedical Engineering, University of Virginia, Charlottesville, Virginia, United States of America; Qingdao Agricultural University, China

## Abstract

In order to develop a medical alternative to surgical ovarian diathermy (OD) in polycystic ovary syndrome (PCOS) more mechanistic information is required about OD. We therefore studied the cellular, molecular and vascular effects of diathermy on the ovary using an established ovine model of PCOS. Pregnant sheep were treated twice weekly with testosterone propionate (100 mg) from day 30–100 gestation. Their female offspring (n = 12) were studied during their second breeding season when the PCOS-like phenotype, with anovulation, is fully manifest. In one group (n = 4) one ovary underwent diathermy and it was collected and compared to the contralateral ovary after 24 hours. In another group a treatment PCOS cohort underwent diathermy (n = 4) and the ovaries were collected and compared to the control PCOS cohort (n = 4) after 5 weeks. Ovarian vascular indices were measured using contrast-enhanced ultrasound and colour Doppler before, immediately after, 24 hours and five weeks after diathermy. Antral follicles were assessed by immunohistochemistry and ovarian stromal gene expression by quantitative RT-PCR 24 hours and 5 weeks after diathermy. Diathermy increased follicular atresia (*P*<0.05) and reduced antral follicle numbers after 5 weeks (*P*<0.05). There was an increase in stromal *CCL2* expression 24 hours after diathermy (*P*<0.01) but no alteration in inflammatory indices at 5 weeks. Immediately after diathermy there was increased microbubble transit time in the ovarian microvasculature (*P* = 0.05) but this was not seen at 24 hours. However 24 hours after diathermy there was a reduction in the stromal Doppler blood flow signal (*P*<0.05) and an increased ovarian resistance index (P<0.05) both of which persisted at 5 weeks (*P*<0.01; *P*<0.05). In the ovine model of PCOS, OD causes a sustained reduction in ovarian stromal blood flow with an increased ovarian artery resistance index associated with atresia of antral follicles.

## Introduction

Polycystic ovary syndrome (PCOS), the most prevalent endocrine disorder in women of reproductive age [1], [2], is the leading cause of anovulatory related infertility [2], [3]. Several treatment options to induce ovulation are available, the first-line treatment being the anti-estrogen clomifene citrate (CC). Although CC is successful at inducing ovulation in approximately 50–80% of cases, pregnancy rates are suboptimal. This is thought to arise due to the anti-estrogenic effects of CC on the endometrium and luteinising hormone surge [4], [5]. Second-line treatment options are used for women who fail to ovulate and/or conceive in response to CC.

Ovulation induction using gonadotrophins is an effective second-line treatment following CC resistance or failure [6]. Indeed it has been reported that follicle stimulating hormone therapy results in higher pregnancy rates than CC treatment [7], [8]. However such treatment is expensive, requires enhanced monitoring to reduce ovarian hyperstimulation and multiple pregnancy rates and it is not available in all secondary care settings. Therefore, in the UK, laparoscopic ovarian diathermy/drilling (LOD) is also recommended as a second-line treatment for anovulatory PCOS by the National Institute for Health and Care Excellence (NICE).

LOD involves puncturing the ovarian surface in several sites, under direct visualisation, either by diathermy or laser [9]. It can be successful at inducing unifollcullar ovulation and pregnancy without the drawback of endometrial thinning, ovarian hyperstimulation and multiple pregnancies [10], [11]. It has the added benefit of being a single treatment that can improve ovulation for many months, and sometimes years, subsequently [12]. However, as an invasive procedure it requires a general anaesthetic and abdominal surgery, which presents increased risks and additional morbidity. We therefore hypothesised that a medical diathermy paradigm, which mimicked the mode of action of surgical diathermy and encompassed the benefits of LOD, without the pitfalls of surgery, would be an advantageous therapeutic option for PCOS.

There are two key requirements needed to develop pharmaceutical strategies to replicate the effects of diathermy on the ovary. The first is a deeper understanding of the acute and chronic effects of surgical diathermy on the polycystic ovary to increase insights into its mechanism of action. Mechanistic insights are lacking as the human ovary is not accessible for *in vitro* investigation following diathermy. The second is a clinically realistic preclinical model of anovulatory PCOS to develop and test novel medical strategies for ovulation induction. We aimed to assess the cellular, molecular and vascular effects of ovarian diathermy on the ovary using a large animal model of PCOS.

Herein we report the effects of ovarian diathermy in an established ovine model of anovulatory PCOS. We used the paradigm of prenatal androgenisation from day 30 to day 100 of gestation in sheep to create offspring who develop a robust PCOS-like condition. We studied these offspring in the second breeding season when the anovulatory PCOS-like phenotype is fully established [13]–[19].

## Materials and Methods

### Ethical Statement

Studies were reviewed by University of Edinburgh Animal Research Ethics Committee and conducted under Project Licence approved by the UK Home Office.

### Reagents

All reagents and chemicals were obtained from Sigma-Aldrich (Dorset, United Kingdom), unless stated otherwise. Progesterone was measured in weekly plasma samples by radioimmunoassay as described previously [20]. This was used to confirm anovulation prior to diathermy and assess the effect of ovarian diathermy on the induction of ovulation.

### Animal treatments

Scottish Greyface ewes (*Ovis aries*) of comparable body condition score were cycle-synchronised using progesterone sponges and mated with Texel rams. Pregnant ewes received intramuscular injections of 100 mg testosterone propionate (TP; AMS Biotechnology Ltd, Abingdon, United Kingdom) in vegetable oil twice weekly, from day (d) 30 to 100 of gestation (d147) and delivered normally. After weaning, the resulting female offspring were housed together in spacious pens and hay was available *ad libitum*, with Excel Ewe Nuts (0.5–1.0 kg/day; Carrs Billington, Lancashire, UK) and Cystalayx Extra High Energy Lick (Caltech Solway Mills, Cumbria, UK). These offspring (n = 12) were maintained until they were adults in their second breeding season (22 months of age) after confirmation of anovulation by serial progesterone assessment for eight weeks prior to randomisation.

The sheep were then randomly divided into three PCOS cohorts (Table 1). The acute cohort (n = 4) were used to investigate the early cellular, molecular and vascular effects of diathermy on the ovary. They underwent mini-laparotomy, ovarian assessment and unilateral ovarian diathermy, allowing the other ovary to serve as an internal control. Twenty four hours later they underwent mini-laparotomy, ovarian assessment and ovarian collection under terminal anaesthesia. The chronic cohort (n = 8) studied the later effects of diathermy on the ovary. A treatment group (n = 4) underwent mini-laparotomy, ovarian assessment and bilateral ovarian diathermy whilst the control group (n = 4) underwent mini-laparotomy and ovarian assessment without diathermy. Five weeks later they underwent mini-laparotomy, ovarian assessment and ovarian collection under terminal anaesthesia. The study was completed in the last month of the breeding season when ovulation would still be expected.

**Table 1:** Summary of treatment and investigation including contrast enhances ultrasound (CEUS) and reverse transcription polymerase chain reaction (RT-PCR).

	Acute (n = 4)		Chronic (n = 4)		Chronic (n = 4)	
	Ovary 1	Ovary 2	Ovary 1	Ovary 2	Ovary 1	Ovary 2
	Diathermy	Control	Diathermy	Diathermy	Control	Control
**Pre-Treatment**						
CEUS	✓	✓				
Doppler	✓	✓	✓	✓	✓	✓
Resistance Index	✓	✓	✓	✓	✓	✓
**Post- Immediate**						
CEUS	✓	✓				
**Post- 24 hours**						
CEUS	✓	✓				
Doppler	✓	✓				
Resistance Index	✓	✓				
Histology	✓	✓				
RT-PCR	✓	✓				
**Post- 5 weeks**						
Doppler			✓	✓	✓	✓
Resistance Index			✓	✓	✓	✓
Histology			✓	✓	✓	✓
RT-PCR			✓	✓	✓	✓

### Diathermy protocol

A mini-laparotomy was performed with sterile technique under general anaesthesia, induced using isoflourane (Isoflo, Abbott Animal Health, Maidenhead, UK). A midline incision exposed the ovaries that were stabilised with a clamp to the uterine fundus. Ovarian diathermy was performed using a needle probe with monopolar coagulation current (Surgitran, STW-100). The coagulation current was applied to the ovary at four separate sites of approximately 4 mm in depth for 10 seconds. Ovarian blood flow and ovarian artery resistance index were assessed prior to and after diathermy. Following surgery, all sheep received antibiotics (Penicillin and Dihydrostreptomycin), analgesics and they were carefully monitored during their rapid recovery.

### In vivo measurement: Contrast enhanced ultrasound (CEUS) of ovarian capillary bed

All ultrasound imaging was undertaken using a Philips iU22 ultrasound scanner (Philips Medical Systems, Bothwell, WA, USA) with linear array transducers L9-3 and L15-7. Using microbubbles along with ultrasound scanning provides a novel method to assess the capillary bed of the ovary. A well-established house contrast agent [21] was utilised. Briefly decafluorobutane microbubbles were prepared by a standard sonication protocol from an aqueous micellar dispersion of phosphatidylcholine (2 mg/ml; Avanti Lipids) and PEG stearate (2 mg/ml; Stepan Kesso). Following two days incubation under fluorocarbon atmosphere in the refrigerator, microbubbles floated to the top of the reaction vessel forming a thick cake. Infranatant containing micellar lipid was removed and replaced with degassed perfluorocarbon-saturated saline. Larger bubbles (in excess of 8–10 µm size) were removed from the preparation by flotation in normal gravity. Microbubbles in aqueous saline were then packaged in glass vials with Teflon-lined rubber stoppers and sealed under perfluorocarbon atmosphere. This preparation has a concentration of 10^9^ microbubbles per ml with a 2 µm average size.

A bolus (0.2 ml volume) was injected into a jugular vein catheter followed by a 10 ml saline flush to ensure all contrast agent was administered. This protocol provides good contrast in the ovary while avoiding signal saturation at peak contrast. CEUS was carried out in sheep destined for the acute analysis. The wash in time (WIT) was recorded for contrast before diathermy, immediately after diathermy and 24 hours after diathermy in triplicate. This is considered a reproducible measurement in the literature [22]–[24]. Details of the protocol for calculating the WIT under these conditions have been reported elsewhere [24].

### In vivo measurement: Ovarian Blood Flow

The whole ovary was scanned in the longitudinal plane in colour Doppler mode with fixed thresholding and video images stored. Doppler scans were replayed back on the quantification software (Q-LAB v6, Philips Healthcare, Andover, MA, USA) and three images per ovary with the greatest area of blood flow were chosen for quantification by two observers blinded to the treatment groups. Using Image J software (http://rsbweb.nih.gov/ij/) the area of total Doppler signal flow for each image was quantified and averaged per ovary.

### In vivo measurement: Resistance Index

The ovarian artery was identified using colour Doppler assessment with an L15-7 ultrasound probe. Resistance index (RI) of the ovarian artery was assessed using automatic RI measurements calculated on the Philips iU22 Ultrasound system backed up with manual measurement confirmation. A continuous wave of at least five peaks was obtained and three separate measurements were taken to give an average value. This was repeated three times and the mean of the averaged values per ovary used for analysis.

### Tissue Collection

At the end of the study ewes were sacrificed. Ovaries were collected and cut longitudinally into two sections (two thirds and one third of the ovary). The smaller section was snap frozen and stored at −80°C for subsequent dissection of an area of stroma (1 mm^3^), from the inner aspect of the outer third at least 1 mm from the ovarian surface, with no visible antral follicles, for RNA extraction and gene analysis studies. The larger, two third portion of the ovary was fixed in Bouins solution for 24 hours before transferral to 70% ethanol for paraffin wax embedding, sectioning and immunohistochemical study.

### Immunohistochemistry

Ovarian sections (5 µm) from the middle of the ovary were analysed. A representative section with maximal ovarian diameter, informed by our previous study on the assessment of follicle number in the ewe [20], underwent haematoxylin and eosin (H&E) staining for subsequent antral follicle counting. Immunohistochemical analysis was also performed for markers of cell proliferation (Ki67) and apoptosis (activated caspase 3). Ovarian tissue sections were dewaxed and rehydrated. Sections then underwent antigen retrieval in a decloaking chamber (Biocare Medical, Concord, California) containing sodium citrate retrieval buffer (0.01M, pH 6.0). Peroxidase quenching and blocking steps were performed via incubation in 3% H_2_O_2_ for 10 minutes, avidin and biotin blocking (Vector Laboratories Ltd, Peterborough, United Kingdom) and finally 5% BSA diluted in 20% normal serum from the host species of the secondary antibody. Primary antibodies, diluted in appropriate serum were applied to sections overnight at 4°C. After washing, secondary antibody was applied to slides for 1 hour, followed by Vectastain ABC Elite tertiary complex (PK-1600 series; Vector Laboratories) for 1 hour. Binding was visualised with 3,3′-diaminobenzidine (Dako, Cambridge, United Kingdom) for 30 seconds. Sections were counterstained with hematoxylin and mounted. Negative controls consisted of either primary antibody incubated with a blocking peptide or, in the absence of a specific blocking peptide, non-immune serum of equivalent immunoglobulin concentrations.

### Analysis of tissue sections

Two independent examiners, blinded to treatment, counted the number of follicles with a clear fluid-filled antrum (≥500 µm) from a mid-section of the ovary [20]. The average antral follicle count per ovarian section was recorded. Immunohistochemical staining of whole ovary sections stained for proliferation (Ki67) and atresia (activated caspase 3) were blindly examined by two independent expert examiners. Each antral follicle was examined and staining was divided into two classifications, positive (clearly positive immunostaining present in multiple cells) and negative (scant/absent immunopositive cells). Number of follicles per classification was used for proportional analysis.

### Quantitative Real Time (qRT) PCR

RNA was extracted from tissue using RNeasy mini spin columns following manufacturer’s protocol and concentration measured using NanoDrop 1000 Spectrophotometer. Complimentary DNA (cDNA) was synthesised from 200 ng RNA in accordance with manufacturer’s protocol (Applied Biosystems, California, USA). Subsequently, qRT-PCR was performed using SYBR Green as previously described [25]. Primer3 Input version 0.4, online software, was used to design forward and reverse primers (Table 2) from DNA sequences obtained from Ensembl Genome Browser, sequences were checked for specificity using Basic Local Alignment Search Tool and validity confirmed as previously described [26]. Real-time PCR reactions were carried out in duplicate 10 µl reactions, negative controls consisted of cDNA reaction without reverse transcriptase and a reaction replacing cDNA with nuclease-free water. Melt curve analysis revealed a single amplicon in all cases. *GAPDH* has been reported as a suitable internal control for ovarian stromal gene expression [27] and target gene expression was analysed relative to *GAPDH* and quantified using the ΔCt method.

**Table 2:** List of the primer pairs used in SYBR Green quantitative real-time PCR.

Gene	Forward Sequence (5′– 3′)	Reverse Sequence (5′–3′)
***GAPDH***	GGCGTGAACCACGAGAAGTATAA	AAGCAGGGATGATGTTCTGG
***IL1***	CAGCCGTGCAGTCAGTAAAA	GAAGCTCATGCAGAACACCA
***IL6***	AAATGACACCACCCCAAGCA	CTCCAGAAGACCAGCAGTGG
***IL8***	CAGCAGAGCTCACAAGCATC	TGTGGCCCACTCTCAATAACT
***IL10***	CCAGGATGGTGACTCGACTAGAC	TGGCTCTGCTCTCCCAGAAC
***TNF***	GGTGCCTCAGCCTCTTCT	GAACCAGAGGCCTGTTGAAG
***CCL2***	GCTCCCACGCTGAAGCTTGAAT	GATTGTCTTTAGACTCTTGGGTTGTGGAG
***SOCS3***	CTTCAGCTCCAAGAGCGAGT	ACGCTGAGGGTGAAAAAGTG
***VEGF***	TCTTCAAGCCATCCTGTGTG	TGCATTCACATTTGTTGTGC

### Statistical analysis

Proportional analysis was conducted using a Fishers exact test. The means of two groups were compared with non-paired or paired t-test where appropriate when the data was parametric with equal variance and the Mann Whitney test when not. Where the distribution was not normal logarithmic transformation was used prior to statistical testing. A *P* value of less than 0.05 was considered statistically significant.

## Results

### The effect of diathermy on antral follicles

**Figure 1:**
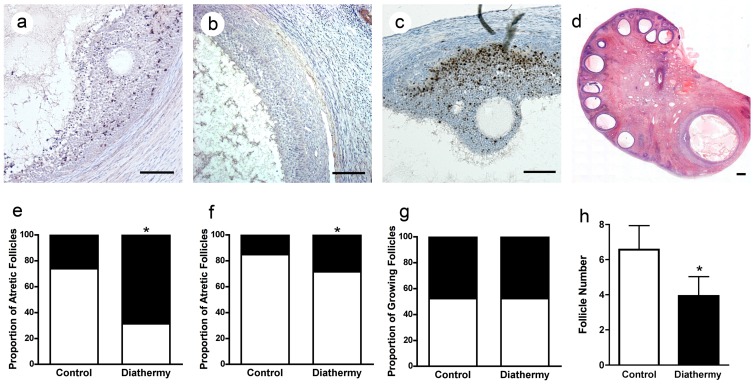
The effect of ovarian diathermy on antral follicles. Immunohistochemistry for activated caspase 3 (brown) in antral follicle showing positive staining (a) and negative staining for atresia (b). (c) Immunohistochemistry for Ki67 showing positive staining for proliferation (brown) in an antral follicle. d) H&E stained mid ovarian section showing antral follicles. The proportion atretic follicles (black) and healthy follicles (white) in control and diathermy ovaries after 24 hours (e) and 5 weeks (f). (g) The proportion of follicles positive (black) and negative (white) for Ki67 (black) proliferation marker (g) and the number of antral follicles (h) in control and diathermy ovaries after 5 weeks. *P<0.05. Scale bar = 100 µm (a–c), 1 mm (d).

We first assessed whether diathermy could induce atresia of antral follicles. Using immunostaining for activated caspase 3 as a marker of atresia each follicle in the tissue section was classified as positive (Fig. 1a) or negative (Fig. 1b) for atresia. The proportion of intact antral follicles with marked caspase 3 immunostaining increased more than four-fold (*P*<0.05) 24 hours after diathermy (Fig. 1e). Five weeks after diathermy the proportion of atretic follicles was less (Fig. 1f) but there was still a significantly increased proportion of follicles undergoing atresia (*P*<0.05). We then investigated whether diathermy altered follicular proliferation in the long term. Using immunostaining for the proliferation marker Ki67 (Fig. 1c) we classified each follicle as negative or positive. The proportion of antral follicles with marked Ki67 staining was not altered five weeks after diathermy (Fig. 1 g). However the overall number of antral follicles per ovarian tissue section (Fig. 1d) was reduced five weeks after diathermy (*P*<0.05) (Fig. 1 h).

### Expression of inflammatory markers in the ovarian stroma

**Figure 2:**
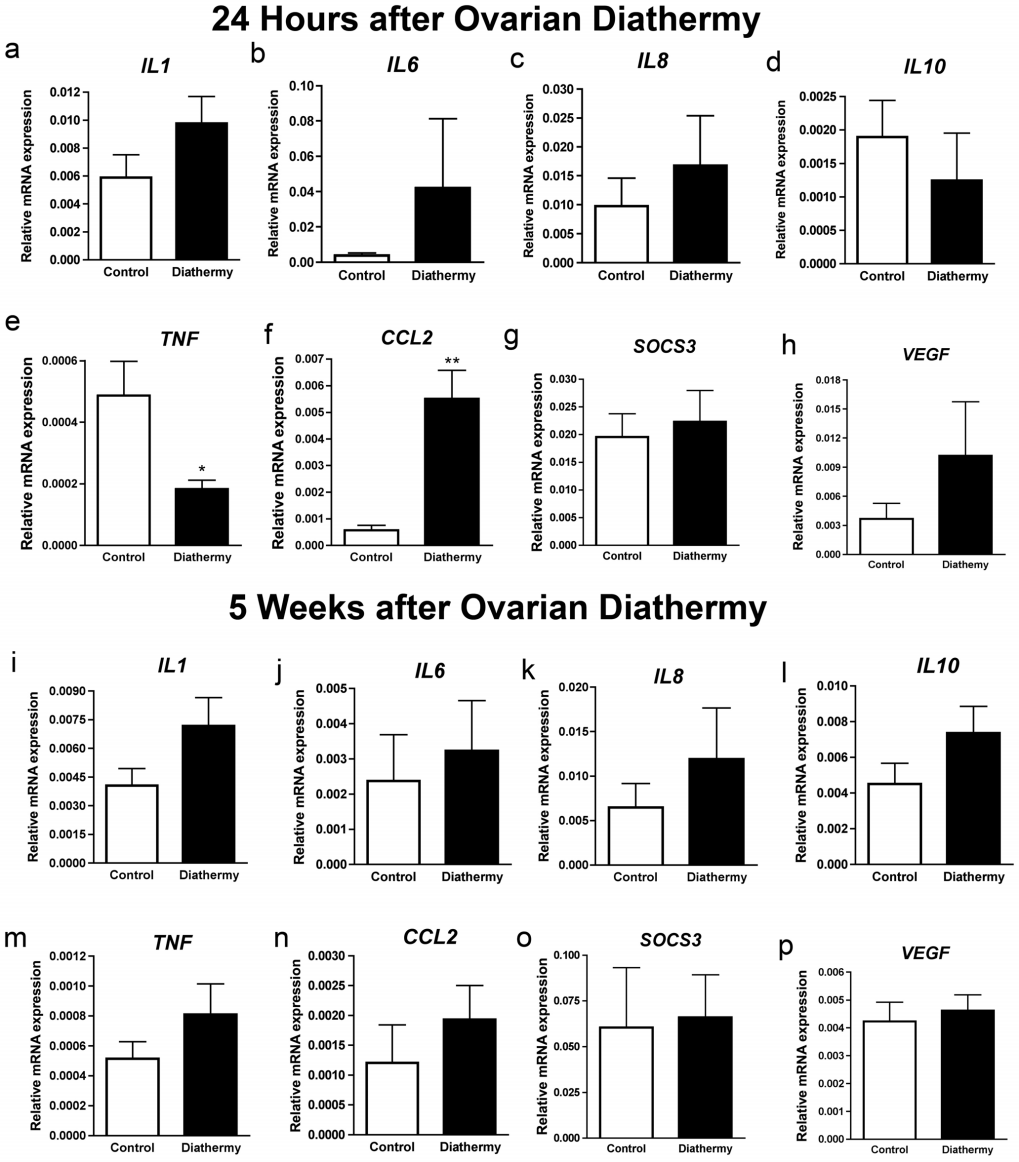
The effect of diathermy on the expression of candidate genes in the ovarian stroma. Twenty four hours after diathermy (n = 4) there was no change in a) *IL1*, b) *IL6*, c) *IL8*, d) *IL10*, g) *SOCS3*, h) *VEGF* when compared to paired control ovaries (n = 4). There was decrease in e) *TNF* and an increase in f) *CCL2* expression. Five weeks after diathermy (n = 4 paired ovaries) there was no difference in the expression of i) *IL1*, j) *IL6*, k) *IL8*, l) *IL10*, m) *TNF*, n) *CCL2*, o) *SOCS3* and p) *VEGF* when compared to control (n = 4 paired ovaries. *P<0.05; **P<0.01.

We next assessed the early and late effects of diathermy on the expression of genes associated with inflammation in the ovarian stroma. Expression of *IL1* tended to increase 24 hours after diathermy but this did not reach statistical significance (*P* = 0.06) (Fig. 2a). At this time point there was no difference in the expression of *IL6* (Fig. 2b), *IL8* (Fig. 2c) or *IL10* (Fig. 2d). However, twenty-four hours after diathermy *TNF* expression was reduced (*P*<0.05) (Fig. 2e) while the macrophage chemokine *CCL2* was up-regulated (*P*<0.01) (Fig. 2f). There was no difference in the cytokine regulator *SOCS3* (Fig. 2 g) or the expression of *VEGF* (Fig. 2 h) 24 hours after diathermy. Five weeks after diathermy there was no difference in *IL1* (Fig. 2i) (*P* = 0.09), *TNF* (Fig. 2 m), *CCL2* (Fig. 2n) or any of the other genes analysed (Fig. 2).

### The effect of diathermy on vascular indices

**Figure 3:**
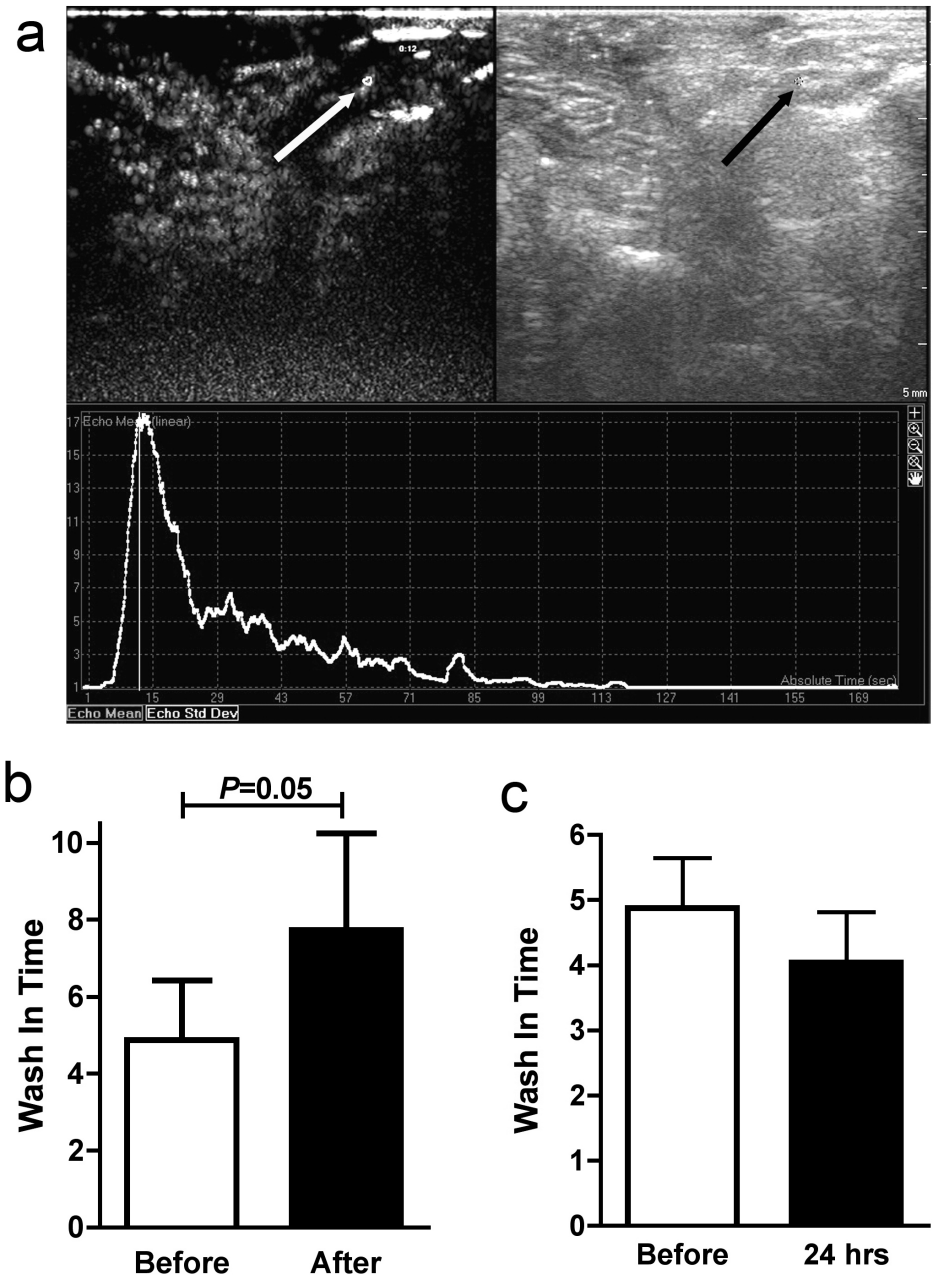
The effect of Diathermy on ovarian stromal microbubble transit time. A) Representative microbubble and greyscale scan with area of interest in the stroma highlighted (arrow) and the transit time graphically illustrated. The wash in time before and immediately after (b) diathermy (n = 4) and 24 hours after (c) diathermy.

**Figure 4:**
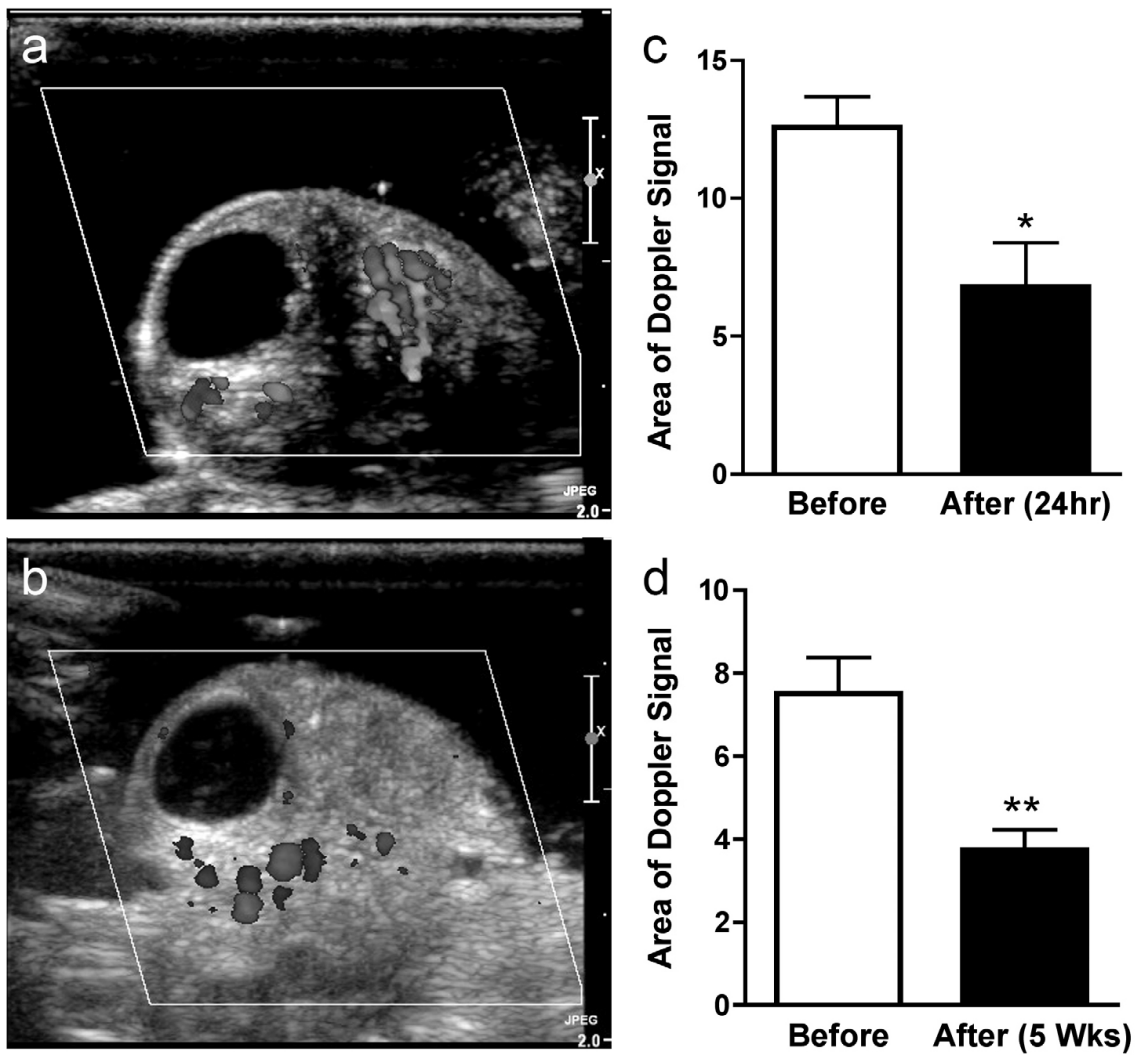
The effect of diathermy on Colour Doppler signal in the ovarian stroma. Representative ovarian scans with fixed threshold Colour Doppler signals visible before (a) and 24 hours after ovarian diathermy (b). Area of fixed threshold Colour Doppler signal in the ovary before and 24 hours (c) and 5 weeks (d) after diathermy. *P<0.05; **P<0.01.

**Figure 5:**
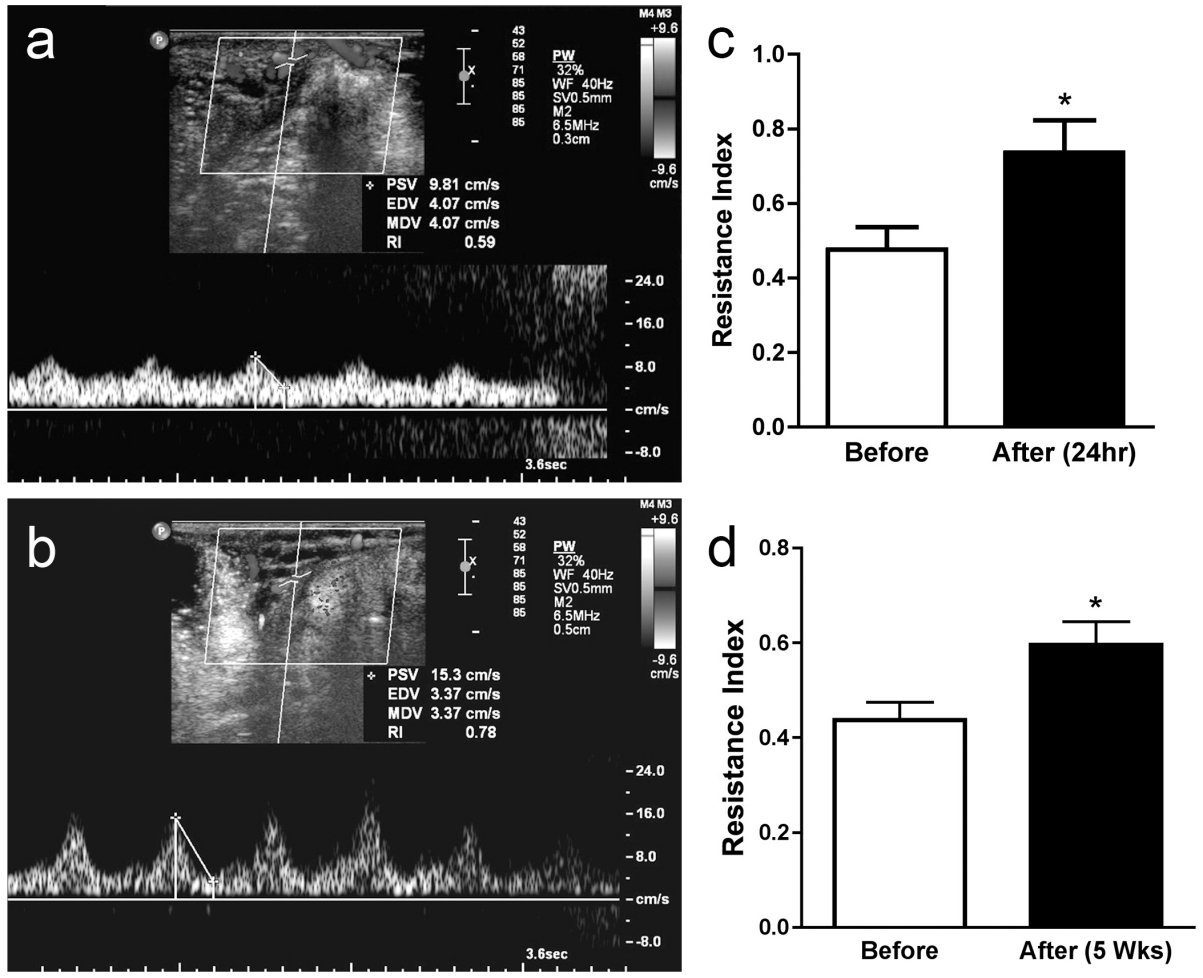
The effect of diathermy on the ovarian artery resistance index (RI). Representative RI scans with Colour Doppler signal wave quantification before (a) and 24 hours after ovarian diathermy (b). Quantified RI of the ovarian artery before and 24 hours (c) and 5 weeks (d) after diathermy. *P<0.05.

In order to assess flow in the ovarian microvasculature we assessed the wash in time (WIT) of sub-capillary sized microbubbles using CEUS (Fig. 3a). Immediately after diathermy there was a strong trend towards increased time for the microbubbles to wash through the ovarian microasculature (Fig. 3b) (*P* = 0.05). However this was not evident 24 hours after diathermy (Fig. 3c). Interestingly there was an effect on larger vessels evident after 24 hours. The area of Doppler flow measurement was quantified in scanned ovaries before diathermy (Fig. 4a) and 24 hours (Fig. 4b) and five weeks after diathermy. There was no difference in coloured Doppler signal area in control ovaries after 24 h hours and five weeks. After diathermy there was a significant reduction in Doppler signal area after 24 hours (P<0.05) (Fig. 4c) that persisted after five weeks (*P*<0.01) (Fig. 4d), suggesting a persistent reduction in vascular volume of larger ovarian vessels. This was associated with an increase in ovarian arterial resistance index (Fig. 5a, b) that was present after 24 hours (*P*<0.05) (Fig. 5c) and persisted at five weeks (*P*<0.05) (Fig. 5d).

### The effect of diathermy on ovarian function

**Figure 6:**
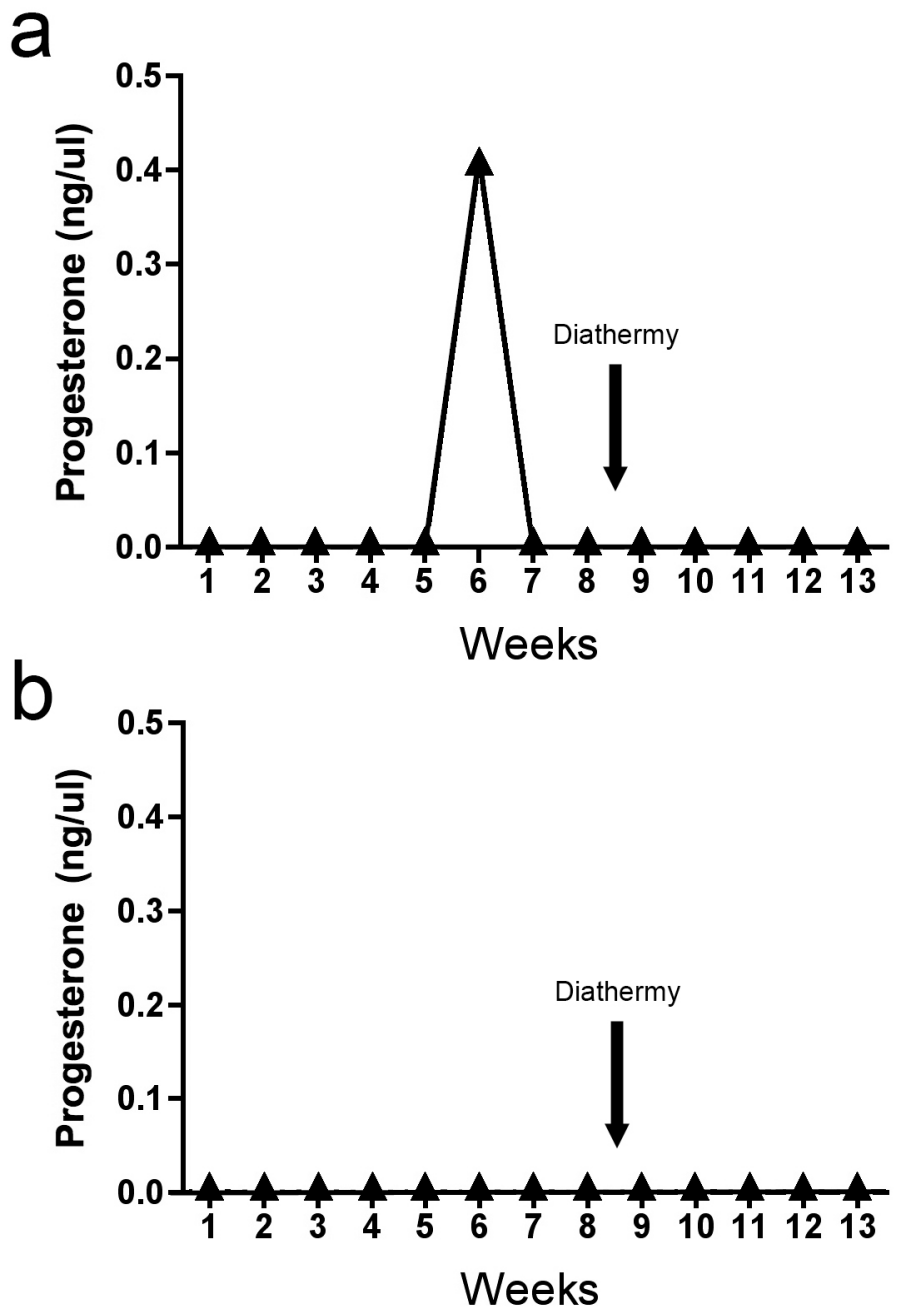
Representative serial plasma progesterone assessment in the sheep with oligoovulation (a) and a representative sheep with anovulation (b). The timing of diathermy is indicated by the arrow.

Serial progesterone measurements were used to determine whether diathermy could induce ovulation in the anovulatory ovine model of PCOS. Although prior to randomisation the sheep were anovulatory, one ovulation was detected in a single sheep before diathermy was conducted (Fig. 6a). Interestingly no sheep ovulated after treatment in either the control PCOS or diathermy PCOS groups (Fig. 6b).

## Discussion

The aim of this study was to identify the cellular, molecular and vascular effects of ovarian diathermy on the ovary in order to inform development of a potential novel therapeutic target to stimulate ovulation in PCOS. We found that diathermy increases follicular atresia and thus decreased the number of antral follicles in the ovary. There is a putative early increase in local inflammation but the most striking findings were the effects on the intra-ovarian vasculature. There were immediate effects on the microvasculature and prolonged effects on the ovarian Doppler signal and arterial resistance index.

A hallmark of PCOS is increased synthesis and secretion of ovarian androgens [1]. The theca cells of the follicle are the source of these androgens and in the polycystic ovary these cells have an enhanced capacity for androgen synthesis [20], [28], [29]. In addition, a polycystic ovary has multiple functional antral follicles that contain a viable oocyte [30], [31] but are not proliferating or undergoing atresia [32], [33]. This means that, in addition to enhanced theca cell function, there is increased theca cell mass in the polycystic ovary. As androgens promote the polycystic ovarian morphology and inhibit the growth of dominant follicles [34], the increased functional mass of theca cells promotes ovarian dysfunction.

It has been proposed that diathermy works by decreasing the load of androgen producing cells within the ovary [35] and our study supports this hypothesis. We observed that increased follicular atresia and thus decreased numbers of antral follicles in the ovary as a consequence of diathermy, thereby reducing theca cell mass within the ovary. In women diathermy can induce systemic hormone changes within days of surgery, including reduced systemic androgen concentrations [12], [36]. A decrease in androgen production would presumably decrease androgen inhibition on follicular maturation beyond the antral stage [12], [34], [36]. Our data would be consistent with one key effect of diathermy at the level of the ovary being the induction of atresia of the accumulated functional follicles.

We wondered if this induced follicular atresia was due to induction of inflammation locally that is detrimental to the health of neighbouring follicles. Diathermy to any tissue induces inflammation and it is therefore unsurprising that we report alterations in the expression of selected inflammatory genes in the ovarian stroma. In a study investigating diathermy in a normal sheep ovary, the authors report an early neutrophil response followed by a macrophage response [37]. We report early increased expression of the macrophage chemotractant *CCL2* [38] that would suggest stimulation of macrophage influx. However TNF, a major macrophage product [39], was significantly reduced suggesting that at 24 hours the inflammatory response was still developing.

In the diathermy study of the normal ovine ovary increased inflammatory processes remained apparent at 19 days post-treatment and was suggested to continue beyond this timeframe [37]. In women, increased ovarian volume post diathermy was attributed to inflammation and as ovarian volume decreased at 3 weeks post-treatment it was suggested that inflammation was resolving at that stage [40]. In our study we could find no evidence of ongoing inflammation at a molecular level after five weeks. This would be consistent with diathermy causing early but transient inflammation within the ovary.

We know that macrophages and macrophage products are involved in removing steroidogenic cells from the ovary. The corpus luteum is a transient steroidogenic gland in the ovary that is removed during luteolysis [41]. This involves an influx of macrophages [42] as well as an increase in chemokines and inflammatory markers [41], [43]. These products can inhibit steroidogenic cell function and survival [44], [45]. It is therefore possible that inflammation itself is responsible for the loss of theca cell mass. However it should be noted that self-limiting inflammation is common in the normal ovary as a part of normal ovarian physiology [46], [47].

It is more plausible that it is the effect on the ovarian blood supply rather than inflammation *per se* that is key to increasing follicular atresia and thus theca cell mass. Ovarian stromal blood supply is increased in women with PCOS [48]–[50]. The ovarian artery resistance index was lower in women with PCOS when compared to matched controls [51], [52] suggesting less impedance to blood flow and ultimately increased ovarian vascularity. It is likely that increased blood supply is important in maintenance of the increased follicular mass of the polycystic ovary.

Using CEUS in the anovulatory ovary for the first time we showed that diathermy had an immediate effect on the microvascular circulation. The wash in time is similar to the mean transit time and proportional to the ratio of volume to flow [23]. Considering the significant reduction of volume, the initial WIT increase implies a significant decrease in flow immediately after diathermy. Further, assuming that the vascular volume is not recovered within 24 hours, the decrease in wash in time shows at least a partial recovery of microvascular flow within 24 hours. Colour Doppler showed there was an effect on the stromal vascular volume, which is derived from bigger vessels, that persists for the five weeks analysed. This is corroborated by the associated increased arterial vascular resistance index, which confirms a persistent reduction intraovarian blood circulation. In women ovarian diathermy has been shown to increase the ovarian artery resistance index [53]. These results are consistent with diathermy having an immediate effect on the vasculature that is maintained for the five weeks of this study.

Thus it might be that targeting the ovarian vasculature would be a possible therapeutic strategy in PCOS. VEGF, a potent angiogenic factor is increased in women with PCOS [50], [54] and may be responsible for the increased vascularisation. Serum VEGF is reduced by diathermy in women [55]. We saw no changes in stromal VEGF expression, which is not surprising as the follicles are the primary source of VEGF in the ovary [56]. Blocking VEGF using VEGF Trap or monoclonal antibodies has utility in blocking angiogenesis in ovarian cancer [57]. In the normal primate ovary it reduces theca cell angiogenesis [58] and induces antral follicle atresia [56]. Indeed we have previously suggested its use as a possible treatment in PCOS [59]. Recently it was reported that VEGF Trap may benefit ovarian function in a rodent model of PCOS [60].

Unfortunately in our model diathermy did not induce ovulation. We did not look in detail at the endocrine changes induced by diathermy in this model as they have been well described in women and were outside the aims of this study. It may be the diathermy strategy or the ovine PCOS model induced by prenatal programming that requires refinement for further study. With regards to the diathermy strategy there is some evidence that the adjustment of thermal dose to ovarian volume may be better that the fixed dose regimen used in this study [61]. There may be an increased hypothalamic pituitary contribution to the anovulatory phenotype in the prenatally androgenised ewe. In this model we used the conventional treatment where prenatal androgens are started at d30 that has a more extreme phenotype [19] than our refined model where androgens are started at d60 gestation [20], [62]. In addition we have not assessed the effect of diathermy on ovaries from ovulatory sheep not programmed to have PCOS. While this has been done previously the effect of diathermy on ovulation was not an outcome of that study [37].

It is however clear that diathermy works best in women who are slim without marked metabolic abnormalities [12], [63], [64]. We and others have shown insulin resistance and other metabolic abnormalities in the ovine PCOS model [17], [26], [62]. It could be therefore speculated that the sheep model parallels the more obese women with PCOS with metabolic dysfunction. Indeed insulin sensitisers improve outcomes in these women with PCOS [65]–[67], and in an ovine PCOS model rosiglitazone prevented the cycle deteriation that is characteristic of these sheep as the breeding season progresses [68].

A medical alternative to surgical diathermy remains attractive. It is possible that targeting the increased ovarian vasculature in PCOS may have utility. However we have shown that the ovine PCOS model does not respond to conventional diathermy by ovulating. This means that a preclinical model to assess novel diathermy-like treatments is not currently established.
